# Supplementary figures and images for: Uukuniemi virus infection causes a pervasive remodelling of the RNA-binding proteome in tick cells
Source: PLoS Pathog. 2025 Aug 4;21(8):e1013393. doi: 10.1371/journal.ppat.1013393 (PMC12342294; doi:10.1371/journal.ppat.1013393)

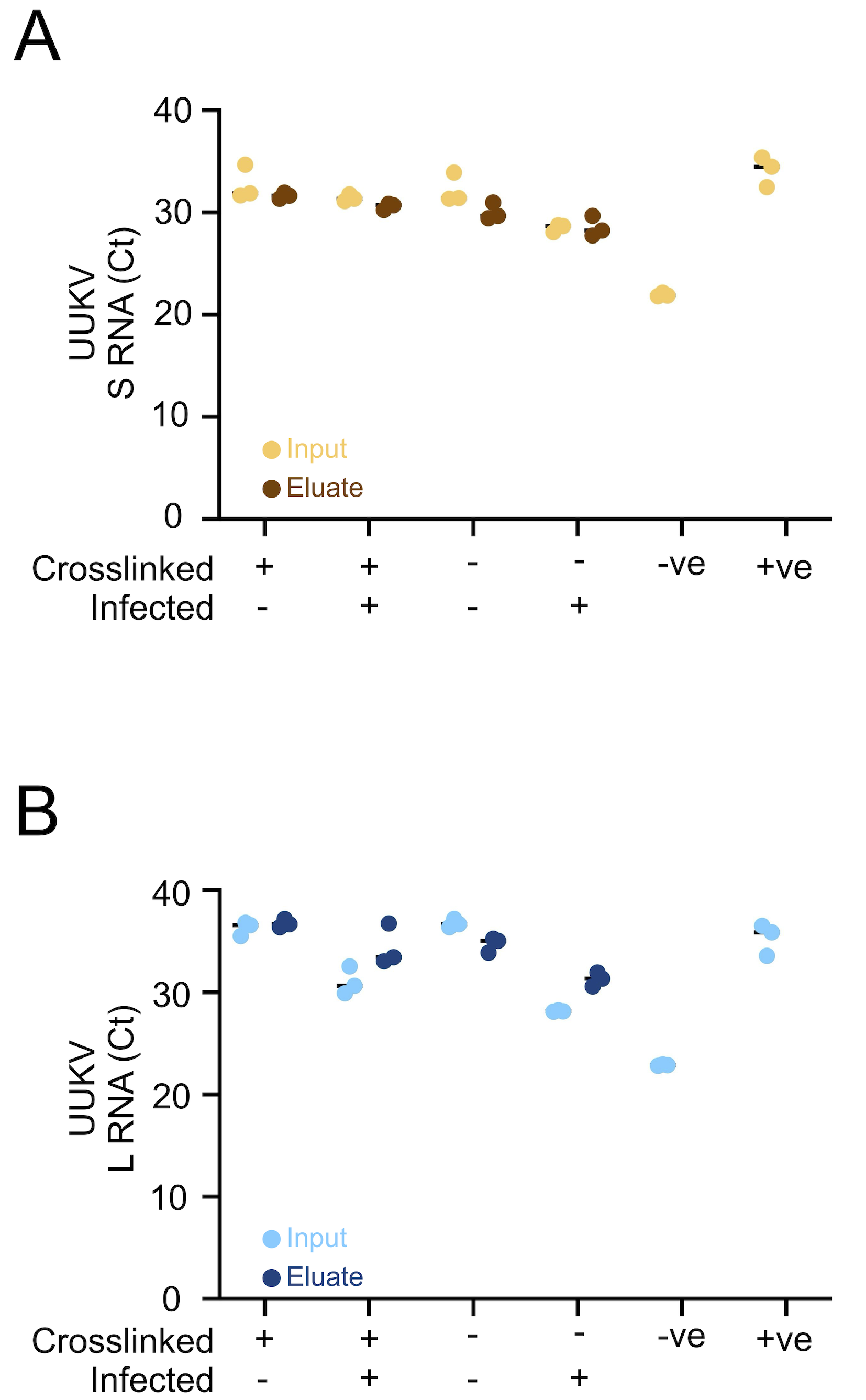

Supplement: S1 Fig — (A) Quantity of UUKV S RNA within input and eluate samples of ISE6 cell monolayers treated by RIC. (B) Quantity of UUKV LRNA within input and eluate samples of ISE6 cell monolayers treated by RIC. (TIFF) [file ppat.1013393.s001.tiff]
