# Supplementary material for: Uukuniemi virus infection causes a pervasive remodelling of the RNA-binding proteome in tick cells
Source: PLoS Pathog. 2025 Aug 4;21(8):e1013393. doi: 10.1371/journal.ppat.1013393 (PMC12342294; doi:10.1371/journal.ppat.1013393)
Supplement: S4 Table — (DOCX) [file ppat.1013393.s008.docx]

**Table S4: Table of oligonucleotide primers used in the production of double stranded RNA, including primer name, sequence and description of use.**

| Primer name | Primer sequence (5’ to 3’) |
| --- | --- |
| eGFP dsRNA Forward | GTAATACGACTCACTATAGGGATGGTGAGCAAGGGCGAGGAGCTGTTC |
| eGFP dsRNA Reverse | GTAATACGACTCACTATAGGGCTGGGTGCTCAGGTAGTGGTTGTCGGGC |
| UUKV N dsRNA Forward | GTAATACGACTCACTATAGGGATGAGACCCTCCCTGAGGAC |
| UUKV N dsRNA Reverse | GTAATACGACTCACTATAGGGATCTGAGGACAGTTGCAGCC |
| AGO2 dsRNA Forward | GTAATACGACTCACTATAGGGACGTGAACAAGACGTCTCCC |
| AGO2 dsRNA Reverse | GTAATACGACTCACTATAGGGGGAGCTCCTTCACCATCGAG |
| PABP1 dsRNA Forward | GTAATACGACTCACTATAGGGGGCTGTTCCCCCTCATCCAC |
| PABP1 dsRNA Reverse | GTAATACGACTCACTATAGGGTCACTCCTTCTTGAGCGAG |
| XRN1 dsRNA Forward | GTAATACGACTCACTATAGGGCAACTGCCGGAAAGGTGTTG |
| XRN1 dsRNA Reverse | GTAATACGACTCACTATAGGGCTGCTTGTTGGGTGGCTTTC |
| TOP3B dsRNA Forward | GTAATACGACTCACTATAGGGCCGTCTACGAGTACATGGGC |
| TOP3B dsRNA Reverse | GTAATACGACTCACTATAGGGGGTAGTCACAGCCTTGACCC |
| SND1 dsRNA Forward | GTAATACGACTCACTATAGGGTTGACTACGGCAATCGGGAC |
| SND1 dsRNA Reverse | GTAATACGACTCACTATAGGGGACCAGCAGGGTCACAAAGT |
| RBM8A dsRNA Forward | GTAATACGACTCACTATAGGGGGAAGGCTGGATCCTGTACG |
| RBM8A dsRNA Reverse | GTAATACGACTCACTATAGGGTGCGGCGATGACTTCTTTTC |
| EIF3A dsRNA Forward | GTAATACGACTCACTATAGGGTGCCACACCGTTCTATCTCG |
| EIF3A dsRNA Reverse | GTAATACGACTCACTATAGGGATTCCTTGATCTCGTCCGGC |
| UNKL dsRNA Forward | GTAATACGACTCACTATAGGGCCTGTACGAGTACCAGGGGG |
| UNKL dsRNA Reverse | GTAATACGACTCACTATAGGGTCCAGGTCCTGTGGCCTAA |
| RUXE dsRNA Forward | GTAATACGACTCACTATAGGGGGACCAGGCCAAAAAGTTCAG |
| RUXE dsRNA Reverse | GTAATACGACTCACTATAGGGCCAAACCTGAATCCGAGCCC |
| CUL1 dsRNA Forward | GTAATACGACTCACTATAGGGATGTGCTGCGGTTCTACACA |
| CUL1 dsRNA Reverse | GTAATACGACTCACTATAGGGATCTCGTAGATGCCCTTGCG |
| PRKRA dsRNA Forward | GTAATACGACTCACTATAGGGCTACATGGGGCTGAAGGAGC |
| PRKRA dsRNA Reverse | GTAATACGACTCACTATAGGGGTGCTGAGCTCGTCTATGGG |
