# Supplementary material for: Uukuniemi virus infection causes a pervasive remodelling of the RNA-binding proteome in tick cells
Source: PLoS Pathog. 2025 Aug 4;21(8):e1013393. doi: 10.1371/journal.ppat.1013393 (PMC12342294; doi:10.1371/journal.ppat.1013393)
Supplement: S5 Table — (DOCX) [file ppat.1013393.s009.docx]

**Table S5. Table of oligonucleotide primers used in qPCR analysis, including primer name, sequence and description of use.**

| Primer name | Primer sequence (5’ to 3’) |
| --- | --- |
| UUKM Standard Forward | ACTTGGCATCTGCCACCATGTTAATC |
| UUKM Standard Reverse | GCCGACCCACACAAAGACA |
| UUKM qPCR Forward | TGCTACTTTCGGTGCCCTAA |
| UUKM qPCR Reverse | CAGGAGGCTTTGAACCAACC |
| UUKS Standard Forward | AGGCTCTATCTGCTGGAGTTCTCC |
| UUKS Standard Reverse | TTCTTCACTGCAATTGAGCAAGCACC |
| UUKS qPCR Forward | TTGGTGAATGTGGTGTCCAG |
| UUKS qPCR Reverse | GCGTTATGGGATGAAGAAGAAG |
| UUKL Standard Forward | GGATTCAAGTACAATGTCTGGGTGG |
| UUKL Standard Reverse | CAGGCTCACTTCAAAGCCATCTCGG |
| UUKL qPCR Forward | TGCTGCTCCAAAGAAAACCT |
| UUKL qPCR Reverse | TTCAACCTGTCCACCAGTCTC |
| 18s Standard Forward | CGTAGTTCCGACCATAAACGA |
| 18s Standard Reverse | CATCTAAGGGCATCACAGACC |
| 18s qPCR Forward | GACTCAACACGGGAAACCTC |
| 18s qPCR Reverse | TAACCAGACAAATCGCTCCAC |
| AGO2 qPCR Forward | CGAGAGCGGGAGATCAACAA |
| AGO2 qPCR Reverse | GAATGCGACCTCGTACCTCC |
| PABP1 qPCR Forward | ACATGATCACTCGCCGATCC |
| PABP1 qPCR Reverse | TCGGCTTGTTCTTGATGGCA |
| XRN1 qPCR Forward | GCTCCGAATCTCTGGACGAG |
| XRN1 qPCR Reverse | CGCCCGAAAAAGTGACTTGG |
| TOP3B qPCR Forward | GCGTGGAGGCTGTACGATTA |
| TOP3B qPCR Reverse | CCCGGATTGATGACGCTCTT |
| SND1 qPCR Forward | GACAACGGTCACTGGAGGTT |
| SND1 qPCR Reverse | GCGGAGTAGTCCTTCCACAG |
| RBM8A qPCR Forward | CTGAAGGAACGAGCTCGGAA |
| RBM8A qPCR Reverse | TTCATCCGTGTCCATGGCTT |
| EIF3A qPCR Forward | ACCCCCTGGAAGAGAGGTG |
| EIF3A qPCR Reverse | ATGTCCCTCGAACGGTCCAT |
| UNKL qPCR Forward | TACGACGAGACGACGGGTAT |
| UNKL qPCR Reverse | GCCCGTCTTGTAGTAACGCA |
| RUXE qPCR Forward | CTACAGAACAGGGCTCGGATT |
| RUXE qPCR Reverse | GCTTCCGCTGCTTAGTCTTTG |
| CUL1 qPCR Forward | GAGGCCAGCATGATCTCCAA |
| CUL1 qPCR Reverse | AAGGGCCTCTTCGGAGTTTG |
| PRKRA qPCR Forward | TTTGGCATCTCTTGCCTCGT |
| PRKRA qPCR Reverse | ATCATCTTGTAGGCAGCCTGG |
